# IL-1 inhibition in Muckle-Wells-Syndrome: withdrawal resulting in rapid deterioration of hearing loss

**DOI:** 10.1186/1546-0096-13-S1-P28

**Published:** 2015-09-28

**Authors:** S Hansmann, K Ambjoernsen, A Koitschev, SM Benseler, JB Kuemmerle-Deschner

**Affiliations:** 1 University Hospital Tuebingen, University Children's Hospital Tübingen, Tuebingen, Germany; 2 University Hospital Tuebingen, University Department of Otolaryngology, Tuebingen, Germany; 3 Hospital Stuttgart, Department of Otorhinolaryngology, Stuttgart, Germany

## Introduction

Muckle-Wells syndrome (MWS), a phenotype within the spectrum of cryopyrine-associated periodic syndrome (CAPS) is characterized by excessive IL-1 release resulting in chronic systemic and organ-specific inflammation including sensorineural hearing loss. During continuous anti-IL-1-therapy clinical symptoms are controlled and hearing loss remains stable. Limited data exists about discontinuation of IL-1-inhibition during the course of disease.

## Objective

To report the case of a MWS patient, with sudden deterioration of hearing loss due to discontinuation of anti-IL-1-therapy and improvement of hearing after re-therapy.

## Case report

A 29 year old female patient was diagnosed with MWS at age 17. Her left ear had been deaf since early childhood. The patient experienced arthralgia, exanthema, fatigue and progressive hearing loss of the right ear during childhood. Anti-IL-1-therapy with Anakinra was started 4 years after diagnosis. In 2007, she was switched to Canakinumab resulting in complete resolution of exanthema and arthralgia and improved fatigue. Hearing loss was stable during therapy as documented by frequent high frequency pure tone assessments (HFPTA).

When she became pregnant, MWS treatment was changed from Canakinumab to Anakinra as suggested by the safety profile. The patient gave birth to a healthy boy, who has a CAPS mutation.

The patient decided to discontinue IL-1-inhibition while breast-feeding. After four months off anti-IL-1-therapy, her hearing had markedly deteriorated: HFPTA demonstrated a decrease of 20-30 dB in frequencies most relevant for speech discrimination, a substantially impairment for a unilateral deaf patient. The patient decided to stop breast-feeding and Canakinumab therapy was immediately re-initiated. Two months later improved hearing was documented with 5-10 dB but still 10-25 dB less than before discontinuation.

## Conclusion

Long-term IL-1-inhibition prevents decline of hearing ability in CAPS. Withdrawal of treatment may result in rapid and marked hearing loss. An early restart of anti-IL-1-therapy may partially reverse hearing loss. This indicates a window of opportunity for reversal of hearing loss by IL-1-inhibition. To our knowledge, this is the first case in which such a close connection between IL-1-inhibition and hearing ability in MWS has been documented. This case report shows the importance of continuation of anti-IL-1-therapy during pregnancy and breast-feeding to reduce the risk of sequel.

## Consent to publish

Written informed consent for publication of their clinical details was obtained from the patient/parent/guardian/relative of the patient.

